# Blood DNA methylation at AMD candidate loci in discordant monozygotic twins

**DOI:** 10.1038/s41598-026-63246-z

**Published:** 2026-07-27

**Authors:** Fabian Kananen, Hannes Bode, Miina Ollikainen, Ilkka Immonen

**Affiliations:** 1 Department of Ophthalmology, University of Helsinki and Helsinki University Hospital, Helsinki, Finland; 2 Department of Ophthalmology, Faculty of Medicine and Health, Örebro University, Örebro, Sweden; 3 Institute for Molecular Medicine Finland (FIMM), Helsinki Institute of Life Science (HiLIFE), University of Helsinki, Helsinki, Finland; 4 Minerva Foundation Institute for Medical Research, Helsinki, Finland

**Keywords:** Biomarkers, Diseases, Genetics, Medical research, Molecular biology

## Abstract

Age-related macular degeneration (AMD) is a leading cause of visual impairment in older adults, with both genetic and environmental factors contributing to disease risk. Epigenetic mechanisms, particularly DNA methylation, may mediate the effects of environmental exposures on disease-relevant genes, yet their role in AMD remains poorly understood. To investigate blood DNA methylation differences associated with AMD severity, we studied 29 monozygotic twin pairs discordant for AMD from the Finnish Twin Cohort. This design controls for genetic background, sex, age, and shared early-life environment. Genome-wide DNA methylation was measured in whole blood using the Illumina HumanMethylation EPIC BeadChip. Analyses were restricted to 263 AMD candidate genes identified through prior genetic, epigenetic, and transcriptomic studies. Of 9,694 analyzed CpG sites, one site within *ESYT1* reached statistical significance after multiple testing correction, but the corresponding methylation difference was below our predefined threshold for biological relevance. No CpG sites met both statistical and biological significance criteria. These findings do not provide evidence for large, systemic DNA methylation differences in peripheral blood at established AMD loci, though subtle effects below the detection limit of the current sample size cannot be excluded.

**Supplementary Information:**

The online version contains supplementary material available at 10.1038/s41598-026-63246-z.

## Introduction

Age-related macular degeneration (AMD) is a leading cause of irreversible visual impairment in older populations and is characterized by progressive degenerative changes affecting the macula^1,2^. Although genetic susceptibility plays a central role in AMD pathogenesis, environmental and lifestyle factors such as smoking also contribute substantially to disease risk^3,4^. This suggests that epigenetic mechanisms may act at the interface between genetic predisposition and environmental exposure^5^.

DNA methylation is a key epigenetic modification involved in gene regulation and has been increasingly studied in complex, age-related diseases^6^. Several DNA methylation studies, including candidate gene analyses and genome-wide methylation profiling, have reported associations between DNA methylation and AMD, using blood as well as ocular tissues including retina and retinal pigment epithelium^7–11^. However, findings across studies have been heterogeneous, and it remains unclear to what extent systemic DNA methylation differences reflect AMD-related biological processes rather than confounding by age, genetic background, or shared environmental factors^12,13^.

Monozygotic twin studies provide a powerful framework for epigenetic research by controlling for genetic variation, sex, age, and early-life exposures^14^. Discordant twin designs are particularly valuable for studying complex diseases such as AMD, as they allow assessment of disease-associated epigenetic differences largely independent of inherited genetic risk. However, strong phenotypic discordance within twin pairs is uncommon, and to our knowledge, twin-based studies of DNA methylation in AMD are very limited, with prior work primarily focusing on allele-specific methylation rather than differential methylation associated with disease status^15^.

The present study aimed to investigate blood DNA methylation differences associated with AMD using monozygotic twin pairs discordant for AMD severity. Given the modest sample size, we employed a candidate gene–based approach focusing on loci previously implicated in AMD through genetic association studies, transcriptomic and expression quantitative trait locus analyses, as well as DNA methylation studies including both candidate gene analyses and genome-wide methylation profiling in blood and ocular tissues. This approach was chosen to prioritize biologically plausible loci while maintaining statistical power. By combining a discordant twin design with stringent statistical and biological effect size criteria, this study sought to evaluate whether robust, biologically meaningful DNA methylation differences at established AMD loci can be detected in peripheral blood.

## Methods

### Twin collection

Fifty-eight individuals (29 monozygotic twin pairs) from the Finnish Twin Cohort with a known discordance in AMD severity were included in this study^16^. The subgroup of Finnish Twin Cohort participants, both concordant and discordant for AMD, who underwent ophthalmological examination have been described in a previous article^17^. In brief, 106 subjects (53 monozygotic twin pairs) from the Finnish Twin Cohort study were recruited for study visits at the Department of Ophthalmology, Helsinki University Hospital between May 2018 and March 2020. Twin pairs were identified from a previous non-ophthalmic study in which one or both twins had self-reported AMD or its precursors. The zygosity of the twins had been determined in earlier research. Descriptive characteristics of the twin pairs included in the present study are shown in Table 1. One twin pair was discordant for smoking status; the co-twin with more severe AMD was a current smoker, while the less affected co-twin had never smoked.

**Table 1:** Descriptive characteristics of the study participants.

Variable	More affected twin	Less affected twin
Individuals, N	29	29
Age, mean (SD; IQR)		
Age (years)	76.1 (SD 2.7; IQR 75.0–77.6)	76.1 (SD 2.7; IQR 75.0–77.6)
Sex, N (%)		
Female	17 (58.6%)	17 (58.6%)
Male	12 (41.4%)	12 (41.4%)
AREDS grade, N (%)		
1 No AMD	0 (0.0%)	14 (48.3%)
2 Early AMD	10 (34.5%)	10 (34.5%)
3 Intermediate AMD	13 (44.8%)	5 (17.2%)
4 Late AMD	6 (20.7%)	0 (0.0%)
Smoking, N (%)		
Never	18 (62.1%)	19 (65.5%)
Former	8 (27.6%)	8 (27.6%)
Current	3 (10.3%)	2 (6.9%)

SD: standard deviation; IQR: interquartile range; AREDS: Age-Related Eye Disease Study.

The study was approved by the Helsinki and Uusimaa Hospital District Medical Ethics Committee (decision number HUS/2241/2016). Written informed consent was obtained from all participants in accordance with the Declaration of Helsinki.

### Phenotypic measurements

At the study visit, all participants underwent dilated color fundus photography and spectral domain OCT imaging (Heidelberg Spectralis, Heidelberg Engineering, Heidelberg, Germany) and answered a structured questionnaire including smoking status.

The CFPs were classified according to AMD grade as suggested by Age-Related Eye Disease Study Report number 6, as described in a previous article^17,18^. The grade of no AMD or normal age-related macular changes (AREDS 1) was defined as the largest drusen size less than a circle with 63 μm diameter and a total area of less than a 125 μm diameter circle. Early AMD (AREDS 2) as intermediate drusen (largest drusen size ≥ 63 μm but < 125 μm) and/or retinal pigment abnormalities consistent with AMD. Intermediate AMD (AREDS 3) as large drusen (⌀ ≥125 μm), or intermediate drusen and a total drusen area of more than 0.241 disc-diameters if soft indistinct and 0.439 disc-diameter if soft distinct, and/or geographic atrophy without involvement of the central macula. Late AMD (AREDS 4) as geographic atrophy with central macular involvement and/or evidence of neovascular AMD (fibrovascular/serous pigment epithelial detachment, serous or hemorrhagic sensory retinal detachment, subretinal hemorrhage, subretinal fibrous tissue or photocoagulation scars from AMD treatment).

The worse eye, i.e. the higher AREDS grade was used to define AMD status of each subject. Discordance within twin pairs was defined as each co-twin having different AMD grades. The distribution of AREDS grades among twin pairs is shown in Table 2.

**Table 2:**
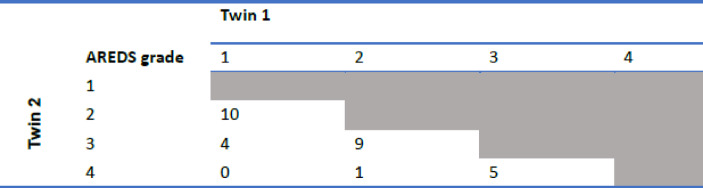
AREDS grade discordance within twin pairs.

### DNA methylation analysis

Whole blood samples were drawn from the participants at the study visit. DNA methylation was assessed using the Illumina HumanMethylation EPIC BeadChip at the Institute for Molecular Medicine Finland Technology Center in 2021, according to the manufacturer’s instructions (Illumina, San Diego, CA, USA). Within each twin pair, samples from the co-twins were placed next to each other on the same array to minimize batch effects within pairs.

All analyses were performed using R programming environment (R version 4.5.1, http://www.r-project.org).

Sample quality control was performed using the R package meffil^19^. All samples passed the quality control metrics.

Probe quality control was performed using the R packages meffil^19^. Probes with bead count < 3 in more than 20% of the samples (2390 probes) or detection p-value > 0.01 in more than 20% of the samples (413 probes) were set to missing. Further, ambiguously mapping and poor-quality probes according to Zhou et al. (negative selection criteria: SNPs with minor allele frequency > 1% near target site, color-channel-switching SNPs, off-target hybridization events, incorrect mapping on the GRCh38 genome) were removed (81565 probes)^20^.

Functional normalization was applied using R package meffil with the sample slide, plate and row as batch variables and including the first 10 principal components of the control probes to remove unwanted between array variability^19^. Afterwards, beta mixture quantile normalization was applied to the normalized data using the R package wateRmelon to further correct the data for probe design bias^21,22^. Supplementary Figs. 1–5 provide QC metrics from the normalization pipeline, including PCA plots and batch variable associations before and after normalization, and the BMIQ probe-design correction plot.

This resulted in a final data set of 781,583 probes. M-values were generated using R package lumi^23^. The Illumina Infinium MethylationEPIC v1.0 B5 manifest file was used for probe annotations.

### Statistical analyses

Statistical analyses were performed using R.

Given the limited sample size, differential DNA methylation analyses were conducted using a candidate gene–based approach. A total of 263 candidate genes were selected to capture genes implicated in AMD through the most comprehensive published genome-wide association studies, transcriptomic and expression quantitative trait locus analyses, as well as DNA methylation studies encompassing both candidate gene analyses and genome-wide methylation profiling, primarily in blood, retina, and retinal pigment epithelium^3,9–11,24–28^. Candidate genes were included if implicated in AMD by at least one of the listed sources, regardless of the consistency of findings across studies. Genes were taken directly from the gene lists reported in the original publications, for GWAS derived sources these were based on chromosomal proximity to index variants. All probes on the EPIC array mapping to the selected genes were included in the analysis. The full list of included candidate genes and sources is provided in supplementary material (Table S1).

Differential DNA methylation was analyzed using a within–pair design restricted to twin pairs discordant for AMD severity. The co-twin with the higher AREDS grade was defined as the more affected twin. This design inherently controls for age, sex, genetic background, and shared early-life environmental factors.

Analyses were performed on M values using empirical Bayes–moderated linear models fitted from the limma package^29^. M-values (logit transformation of β-values) were used for statistical testing due to improved heteroscedasticity properties, while β-values were retained for reporting effect sizes as they are more intuitive and directly interpretable as methylation proportions^30^. Within-pair correlation due to the twin structure was modeled using the duplicateCorrelation function in limma, with twin pair included as a blocking factor. Smoking status (never, former, current) was included as a categorical covariate.

Leukocyte cell-type proportions were estimated using R-package EpiDISH with the centBloodSub reference panel^31,32^. A sensitivity analysis was performed using these cell type proportions as additional covariates alongside smoking status in a secondary limma model.

For each CpG site, mean methylation levels were calculated separately for more and less affected twins. Effect sizes were reported as within-pair differences in β values (more affected minus less affected). Multiple testing correction was performed using the Benjamini–Hochberg false discovery rate (FDR). Differentially methylated CpG sites were defined as those with an FDR-adjusted p value < 0.05 and an absolute mean methylation difference ≥ 5% on the β-value scale.

The methylation difference threshold was applied as secondary criterion for biological relevance, consistent with effect sizes considered meaningful in previous blood-based EWAS^30,33–35^. Given the modest sample size, a threshold substantially above the technical measurement variability of the Illumina EPIC array was considered appropriate to reduce the risk of reporting statistically significant but biologically marginal findings^36^.

A post-hoc power analysis was performed to assess the statistical power of the study. The significance threshold was set at FDR < 0.05, corresponding to an equivalent raw p-value of 3.47 × 10⁻⁶ across the 9,694 tested CpG sites. The per-probe SD of within-pair Δβ was estimated empirically from the limma output, yielding a median per probe SD of 1.68%. With 29 pairs, the minimum detectable effect size was Δβ 1.70% at 80% power. The predefined biological relevance threshold of 5% corresponded to a Cohen´s dz of 2.97, indicating > 99% power to detect effects of that magnitude if present across the analyzed probe set.

During the preparation of this manuscript, the authors used ChatGPT (GPT-5.3, OpenAI) to improve the language of the manuscript. All AI-generated suggestions were reviewed by the authors, who take full responsibility for the content of the published article.

## Results

Differential DNA methylation analyses were performed in 29 monozygotic twin pairs discordant for AMD severity using a candidate gene–based approach. In total, 9,694 CpG sites located within predefined AMD candidate genes were included in the analysis. The top ten CpG sites ranked by nominal p value are summarized in Table 3. Complete differential methylation results for all analyzed CpG sites are provided in the supplementary material (Table S2).

**Table 3:** Top CpG sites associated with AMD discordance ranked by nominal p value.

CpG probe	Gene	Mean β (less affected)	Mean β (more affected)	Δβ (%)	logFC	*P* value	FDR-adjusted *p*
cg00885474	ESYT1	0.974	0.979	+ 0.56	0.340	3.5 × 10⁻⁶	0.034
cg21762107	CTSA	0.013	0.016	+ 0.24	0.248	2.1 × 10⁻⁵	0.104
cg27230984	ABR	0.799	0.783	−1.60	−0.137	9.9 × 10⁻⁵	0.321
cg16252971	RIN3	0.965	0.971	+ 0.63	0.290	2.1 × 10⁻⁴	0.511
cg13996617	LMTK3	0.052	0.042	−0.94	−0.313	4.6 × 10⁻⁴	0.807
cg19253339	SKI	0.941	0.949	+ 0.77	0.214	5.0 × 10⁻⁴	0.807
cg10881923	RPTOR	0.921	0.931	+ 0.99	0.212	6.2 × 10⁻⁴	0.853
cg17232924	SMURF1	0.975	0.978	+ 0.31	0.185	7.0 × 10⁻⁴	0.853
cg07713538	GTF2H4	0.965	0.961	−0.40	−0.158	9.8 × 10⁻⁴	0.972
cg16266672	CCAR2	0.976	0.979	+ 0.23	0.157	1.0 × 10⁻³	0.972

FDR: Benjamini-Hochberg false discovery rate.

**Fig. 1:**
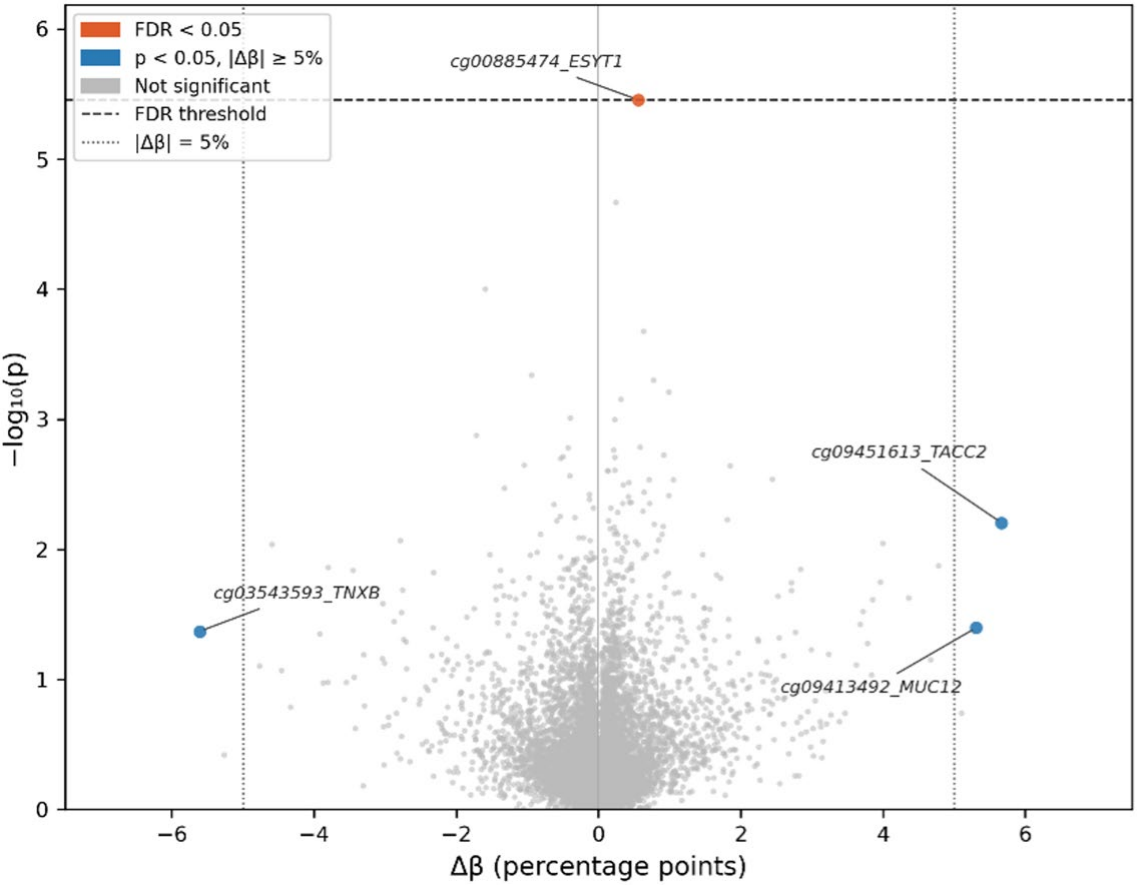
Volcano plot showing nominal p values versus β-value differences between more and less affected co-twins across 9,694 CpG sites. The horizontal line indicates the FDR significance threshold, and vertical lines indicate ± 5% methylation difference.

**Fig. 2:**
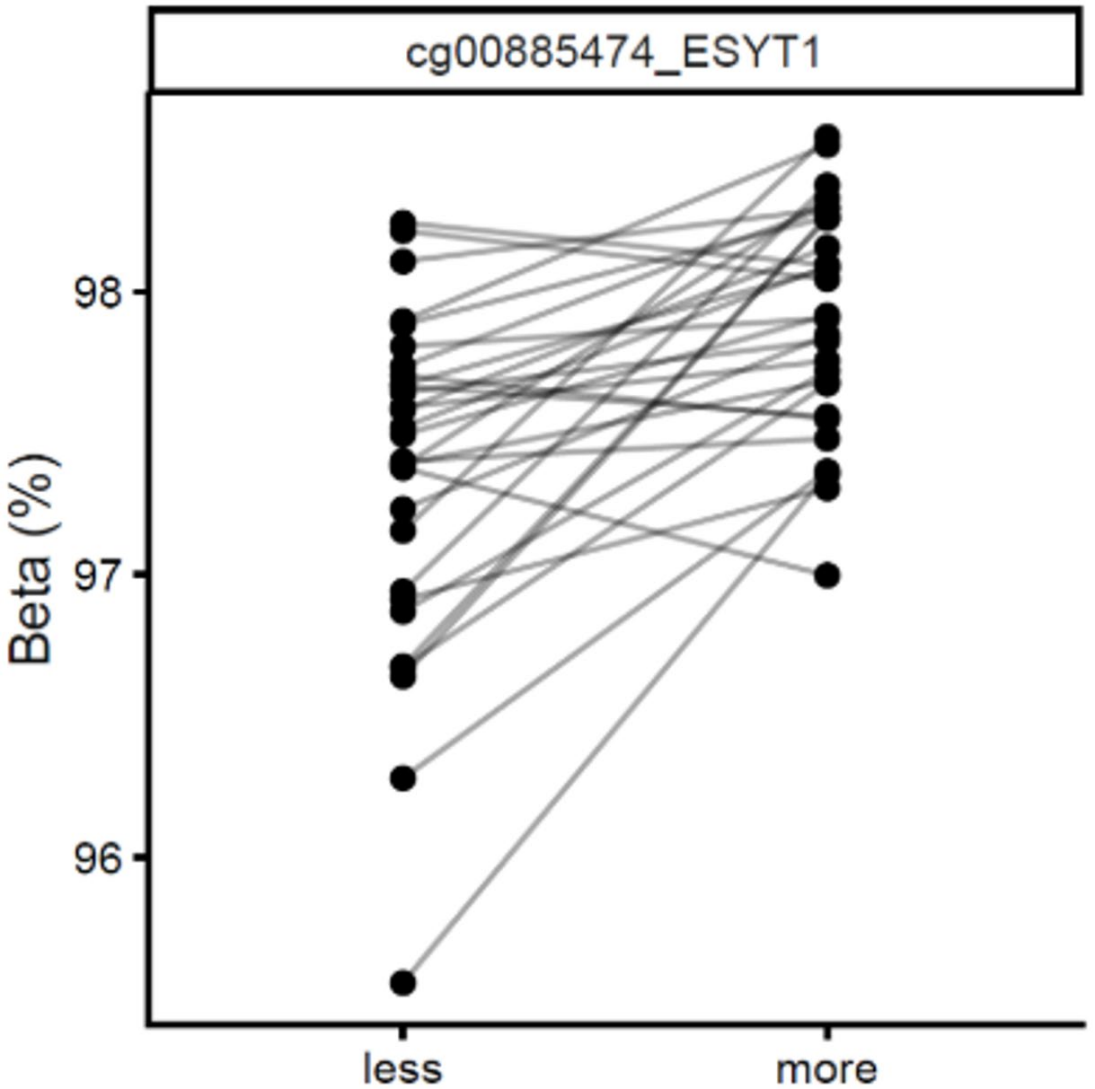
Paired β values for cg00885474 in monozygotic twin pairs discordant for AMD severity, illustrating methylation differences between less and more affected co-twins.

One CpG site, cg00885474 located in the *ESYT1* gene, reached statistical significance after correction for multiple testing (*p* = 0.034). This site showed higher methylation levels in the more affected twins compared with their less affected co-twins (logFC = 0.34). However, the corresponding mean difference on the β-value scale was small (Δβ = 0.56%), below the predefined threshold of 5% used to define biologically meaningful differential methylation. The overall distribution of effect sizes and statistical significance is illustrated in Fig. 1, and paired β-value distributions for cg00885474 are shown in Fig. 2. At this site, within-pair correlation in β values was *r* = 0.35 (*p* = 0.060), reflecting the narrow range of methylation values at this near-uniformly hypermethylated site (mean β ≈ 0.97 in both groups).

No CpG sites met both criteria for differential methylation, defined as an FDR-adjusted p value < 0.05 and an absolute mean methylation difference ≥ 5% on the β-value scale.

Several additional CpG sites within AMD-associated genes showed nominal associations with AMD discordance (uncorrected *p* < 0.05) but did not remain statistically significant after false discovery rate correction. Notably, CpG sites cg09451613 in *TACC2* (Δβ=+5.67%, *p* = 0.006), cg09413492 in *MUC12* (Δβ=+5.31%, *p* = 0.040), and cg03543593 in *TNXB* (cg03543593 Δβ=−5.61%, *p* = 0.043) showed nominal significance and methylation differences greater than 5% but were considered exploratory due to lack of statistical significance after correction for multiple testing.

In the cell-type-corrected sensitivity analysis, no CpG site reached FDR significance. The top-ranked site, cg00885474 in ESYT1, remained the strongest association but was attenuated (FDR-adjusted *p* = 0.105). The three nominally significant CpG sites exceeding the 5% biological relevance threshold cg03543593 in TNXB (*p* = 0.035), cg09451613 in TACC2 (*p* = 0.009), and cg09413492 in MUC12 (*p* = 0.047) remained nominally significant across the models. Full results of the cell-type-corrected analysis are provided in the supplementary material (Table S3).

Overall, these findings indicate limited evidence for robust, biologically meaningful differences in blood DNA methylation at candidate AMD loci within discordant monozygotic twin pairs.

## Discussion

In this study, we investigated blood DNA methylation differences associated with age-related macular degeneration (AMD) using a discordant monozygotic twin pair design and a candidate gene–based approach. By focusing on twin pairs discordant for AMD severity, the analyses inherently controlled for genetic background, sex, age, and shared early-life environmental exposures. Overall, we found limited evidence for robust, biologically meaningful differences in blood DNA methylation at predefined AMD-associated loci.

One CpG site, cg00885474 within the *ESYT1* gene, reached statistical significance after correction for multiple testing. According to Illumina manifest annotation, this probe maps to an exon boundary and gene body region of *ESYT1*, with proximity to transcription start site–associated regions (TSS1500/TSS200) across multiple transcripts. Such genomic localization suggests potential regulatory relevance, as methylation changes in promoter-adjacent or exon boundary regions may influence transcriptional regulation or alternative splicing. *ESYT1* encodes extended synaptotagmin-1, a protein localized at endoplasmic reticulum–plasma membrane contact sites and involved in lipid transfer and calcium-dependent membrane homeostasis^37–40^. These processes are relevant to cellular stress responses and metabolic regulation, mechanisms that have been implicated in retinal aging and degeneration. However, the observed methylation difference on the β-value scale was small (< 1%), falling below the predefined threshold for biological relevance. This indicates that while subtle epigenetic variation at *ESYT1* may be detectable in blood, the magnitude of the effect is unlikely to reflect a major systemic methylation difference associated with AMD.

Several additional CpG sites within AMD-associated genes showed nominal associations together with biologically relevant methylation differences, but none met the predefined criteria for differential methylation after correction for multiple testing. Among the nominally significant sites exceeding the 5% biological relevance threshold, cg03543593 in *TNXB* is of particular interest. Porter et al. (2019) identified a differentially methylated region of hypomethylation within exon 3 of *TNXB* in RPE cells from AMD donors, with concordant reduction in *TNXB* mRNA expression in independent early AMD RPE samples^11^. The present finding of hypomethylation at cg03543593 in more affected co-twins (mean β: 51.0% vs. 56.6%) is directionally consistent with the RPE finding and comes from an independent cohort controlled for genetic background. While this site did not survive correction for multiple testing and is considered exploratory, the directional concordance across independent cohorts and tissues together with the presence of TNX in outer retina, warrants replication in larger studies. *TACC2* and *MUC12* lack prior AMD epigenetic support and are likewise exploratory.

Taken together, these results do not provide evidence for large systemic DNA methylation differences in peripheral blood at known AMD loci, at least within the limits of the current sample size and candidate gene framework. However, blood-based methylation signals may still serve as biomarkers of disease mechanisms occurring in ocular tissues, even if the effects are too small to detect reliably in our dataset.

An additional consideration is the degree of phenotypic discordance within the twin pairs. As shown in the distribution of AREDS grades among discordant pairs (Table 2), most twin pairs differed by only one disease stage, with 10 of 29 pairs discordant between AREDS grades 1 and 2, and 9 pairs between grades 2 and 3. Twin pairs with more pronounced phenotypic differences were uncommon, indicating that large differences in AMD severity are relatively rare within monozygotic twin pairs. This limited phenotypic separation may reduce the ability to detect robust epigenetic differences and suggests that many discordant pairs may represent individuals at different stages along a shared disease trajectory rather than fundamentally distinct disease states. Consequently, observed DNA methylation differences may reflect subtle or transient changes associated with disease progression rather than stable, stage-defining epigenetic alterations.

Cell-type composition was not adjusted for in the primary analysis, as leukocyte proportions have shown high degrees of heritability in MZ twins, implying partial within-pair matching, and including additional covariates in a model with only 29 pairs would reduce available degrees of freedom considerably^41^. In the cell-type corrected sensitivity analysis, the three nominally significant sites with Δβ > 5% (TNXB, TACC2, MUC12) remained nominally significant, while the ESYT1 signal was attenuated (FDR-adjusted *p* = 0.105). The logFC at ESYT1 changed by < 1% between models (0.340 vs. 0.343), suggesting the attenuation of statistical significance more likely reflects reduced power from the additional covariates than true cell-type confounding, though this interpretation is limited by the modest sample size.

Previous studies investigating DNA methylation in AMD have reported heterogeneous findings, with most disease-associated methylation changes being modest in magnitude and often tissue-specific. A recent comprehensive review of epigenetic alterations in AMD summarized differential methylation of genes involved in oxidative stress, immune regulation, lipid metabolism, and extracellular matrix remodeling, including *GSTM1* and *GSTM5* (retina/RPE/choroid), *SKI* (retina/RPE), *GTF2H4* (RPE), and *IL17RC*, *LCAT*, *SIRT1*, *PRSS50*, and global LINE-1 methylation (peripheral blood)^42^. This tissue specificity may partly explain the limited number of biologically meaningful signals observed in the present blood-based analysis, as disease-relevant epigenetic alterations may be more pronounced in ocular tissues than in peripheral blood.

The largely negative findings are important in themselves and may reflect several factors. First, AMD is a tissue-specific disease, and disease-relevant epigenetic alterations may be confined to retinal tissues rather than peripheral blood. Second, epigenetic effects related to AMD may be subtle, cell-type specific, or context dependent, requiring larger sample sizes or single-cell approaches to detect. Third, the candidate gene strategy, while appropriate given the sample size, necessarily limits discovery of novel loci outside established AMD-associated pathways.

Strengths of this study include the monozygotic twin pair design, methylation quality control and normalization according to established protocols, and the use of biologically interpretable effect size thresholds. Limitations include the modest sample size, use of whole blood rather than ocular tissue, and limited power to detect small effect sizes after multiple testing correction.

In conclusion, this twin-based epigenetic study provides limited evidence for robust, biologically meaningful differences in blood DNA methylation at AMD candidate loci. Negative findings may partly reflect the modest sample size, limited phenotypic separation between co-twins, and tissue-specificity of DNA methylation. Future studies integrating larger cohorts, disease-relevant tissues, and multi-omic approaches may be required to clarify the contribution of epigenetic regulation to AMD pathogenesis.

## Supplementary Information

Below is the link to the electronic supplementary material.

Supplementary Material 1

Supplementary Material 2

Supplementary Material 3

Supplementary Material 4

## Data Availability

The data that support the findings of this study are not publicly available due to ethical restrictions and the terms of informed consent under which they were collected. The data contain sensitive personal and medical information from participants of the Finnish Twin Cohort. Requests for data access may be directed to the corresponding author and are subject to approval by the relevant ethics committee and data custodians of the Finnish Twin Cohort.
