# Supplementary material for: Blood DNA methylation at AMD candidate loci in discordant monozygotic twins
Source: Sci Rep. 2026 Jul 27;16:23356. doi: 10.1038/s41598-026-63246-z (PMC13408906; doi:10.1038/s41598-026-63246-z)
Supplement: Supplementary file 4 — Supplementary Material 4 [file 41598_2026_63246_MOESM4_ESM.pdf]

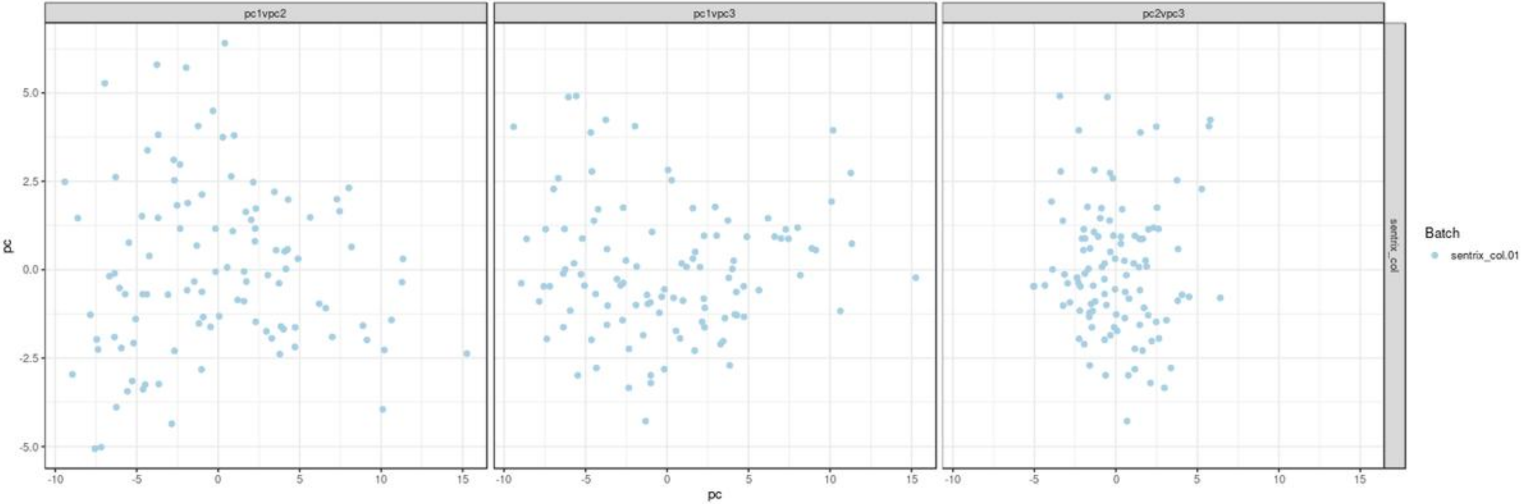

Figure S1a. Principal components of the control probes. The first 3 principal components of the control probe matrix, colored by Slide. Batch variables with more than 10 levels are omitted.

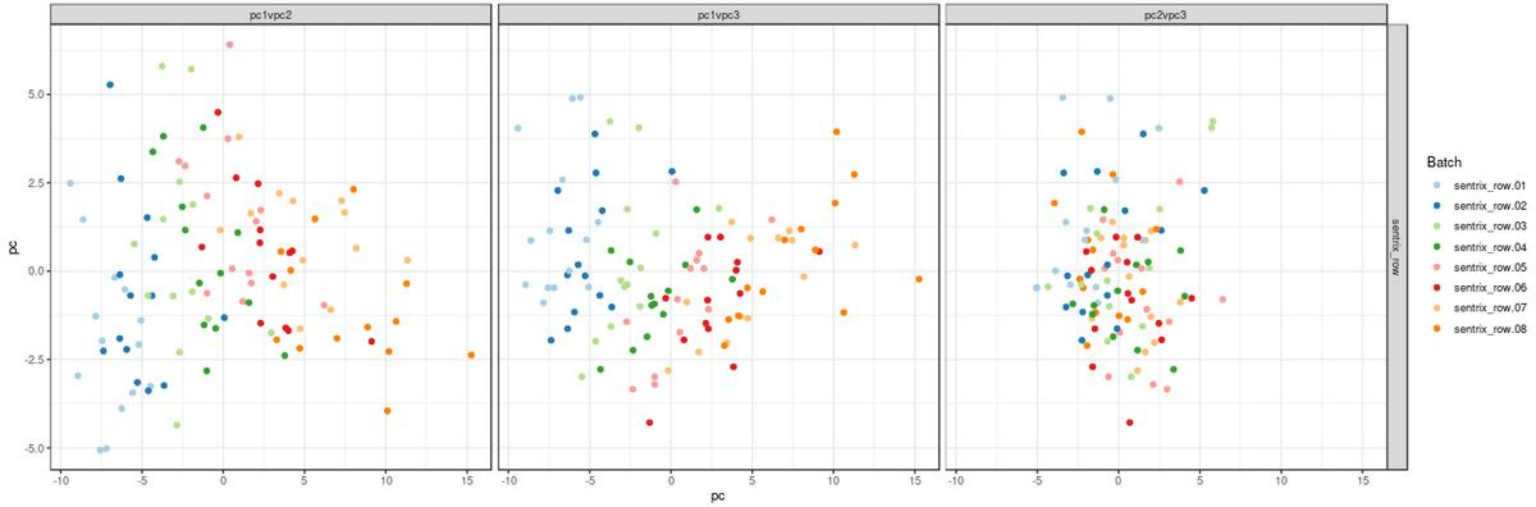

Figure S1b. Principal components of the control probes. The first 3 principal components of the control probe matrix, colored by sentrix_row.

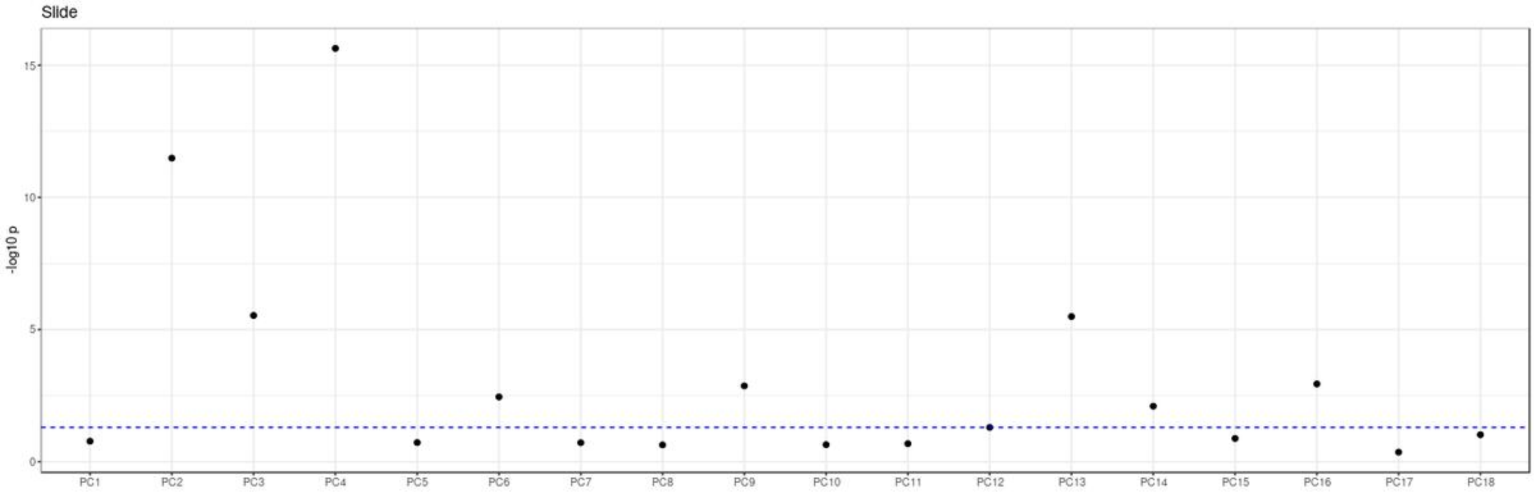

Figure S2a. Control probe associations with measured batch variables (Slide). Principal components of the control probes were regressed against batch variables. Shown are the $-\log_{10}$ p-values for these regressions. The horizontal dotted line denotes p = 0.05 in log-scale.

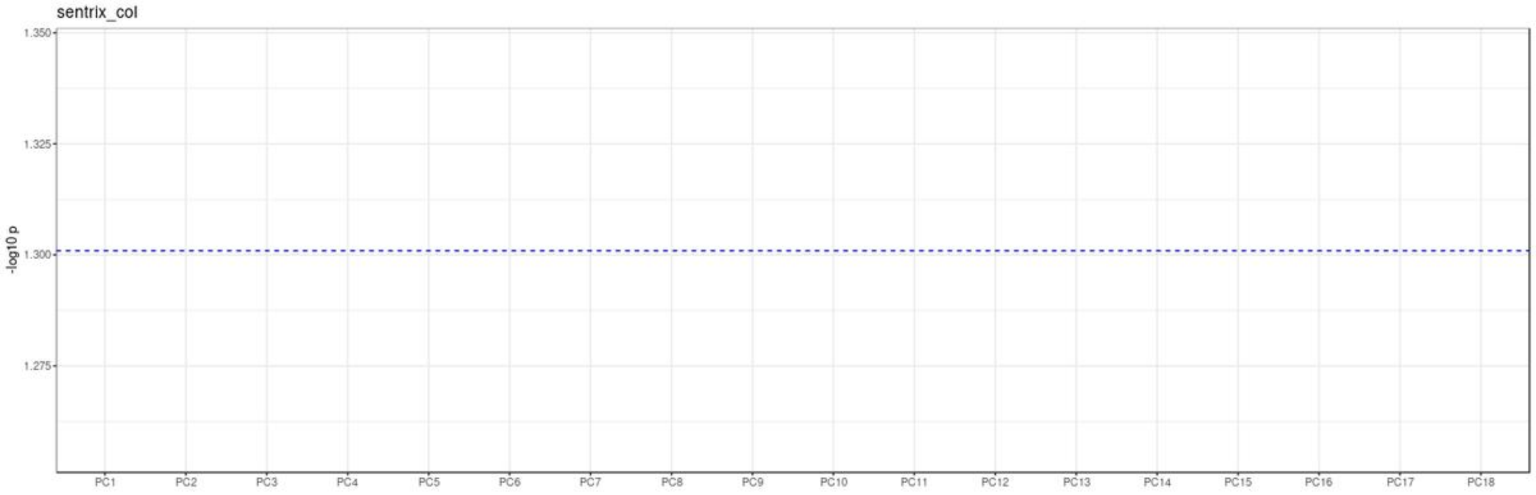

Figure S2b. Control probe associations with measured batch variables (sentrix_col). Principal components of the control probes were regressed against batch variables. Shown are the $-\log_{10}$ p-values. The horizontal dotted line denotes p = 0.05 in log-scale.

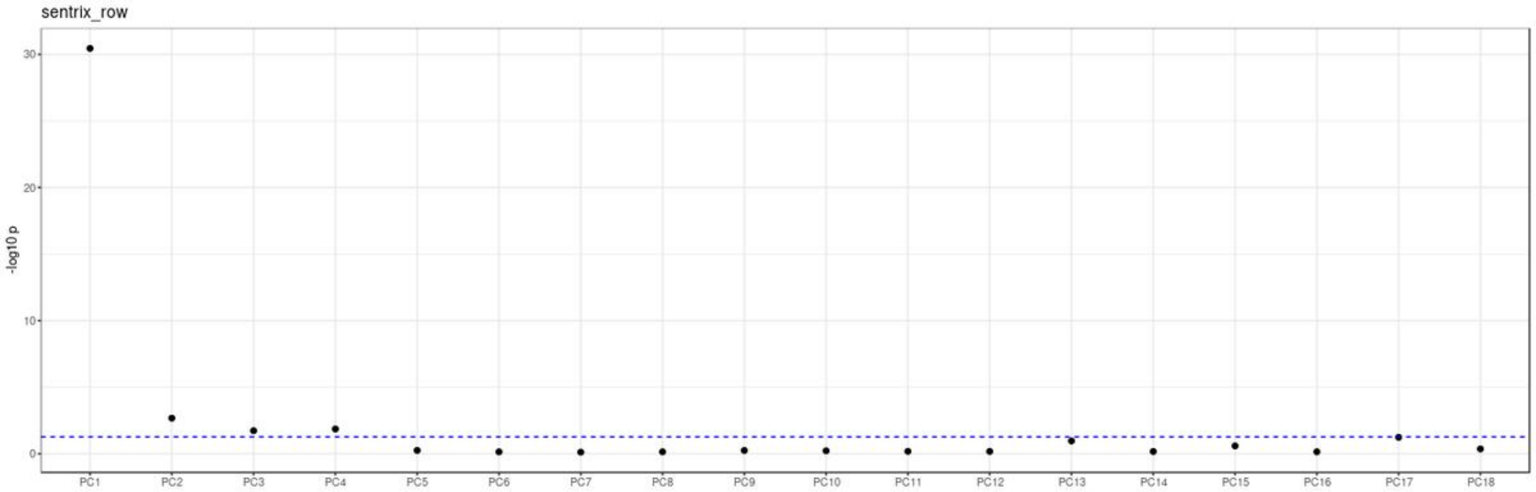

Figure S2c. Control probe associations with measured batch variables (sentrix_row). Principal components of the control probes were regressed against batch variables. Shown are the $-\log_{10}$ p-values. The horizontal dotted line denotes p = 0.05 in log-scale.

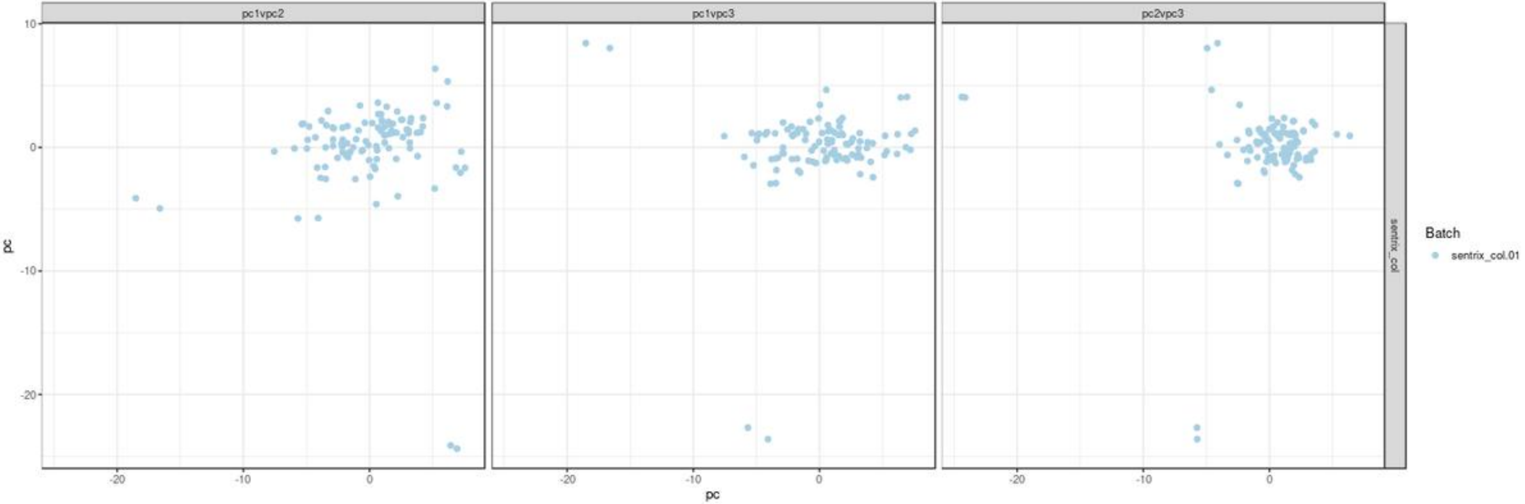

Figure S3a. Principal components of the normalized betas. The first 3 principal components of the most variable normalized probes, colored by Slide. Batch variables with more than 10 levels are omitted.

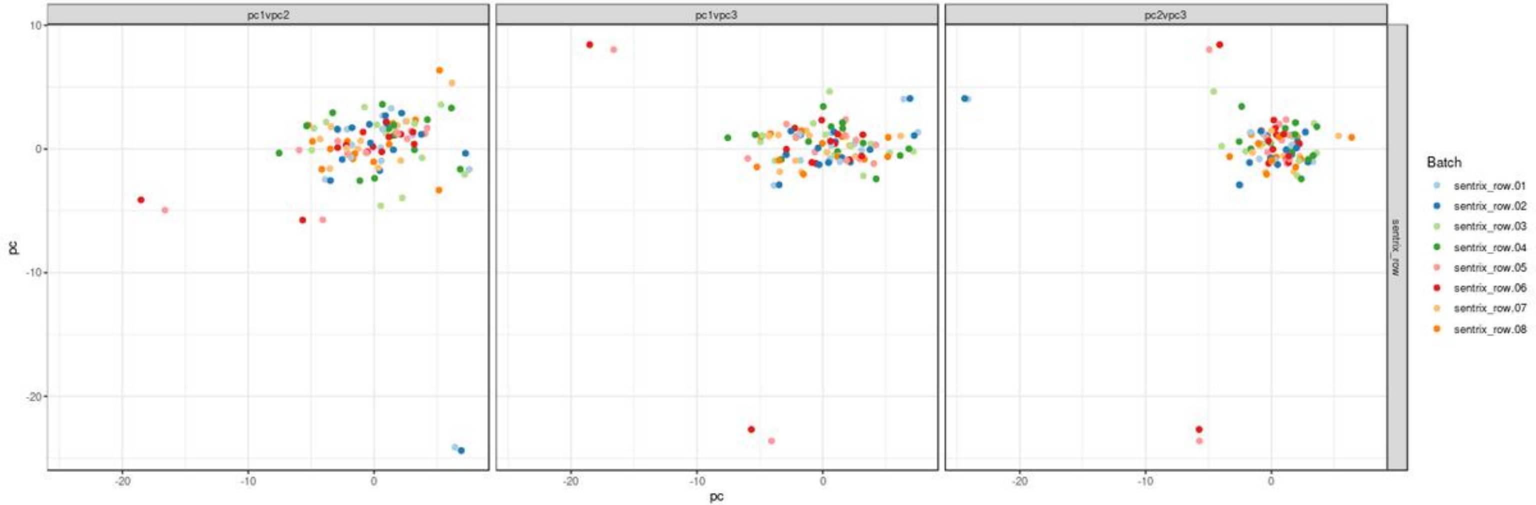

Figure S3b. Principal components of the normalized betas. The first 3 principal components of the most variable normalized probes, colored by sentrix_row.

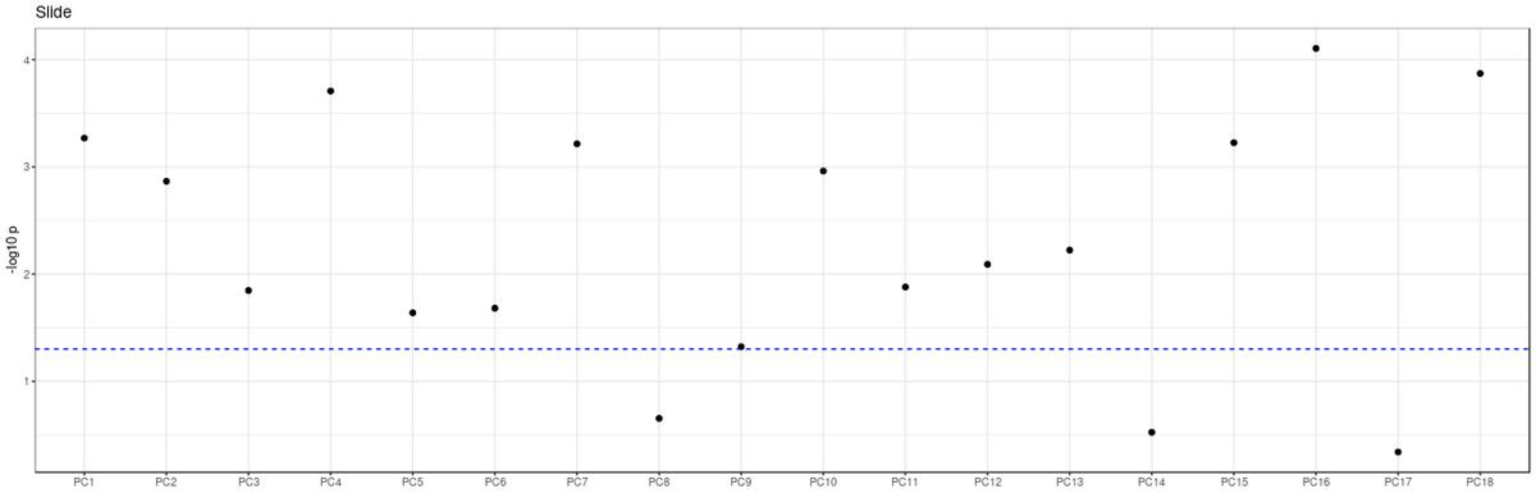

Figure S4a. Normalized probe associations with measured batch variables (Slide). The most variable normalized probes were extracted, decomposed into principal components, and each component regressed against each batch variable. If normalization has performed well there will be no associations between normalized probe PCs and batch variables. Horizontal dotted line denotes p = 0.05 in log-scale.

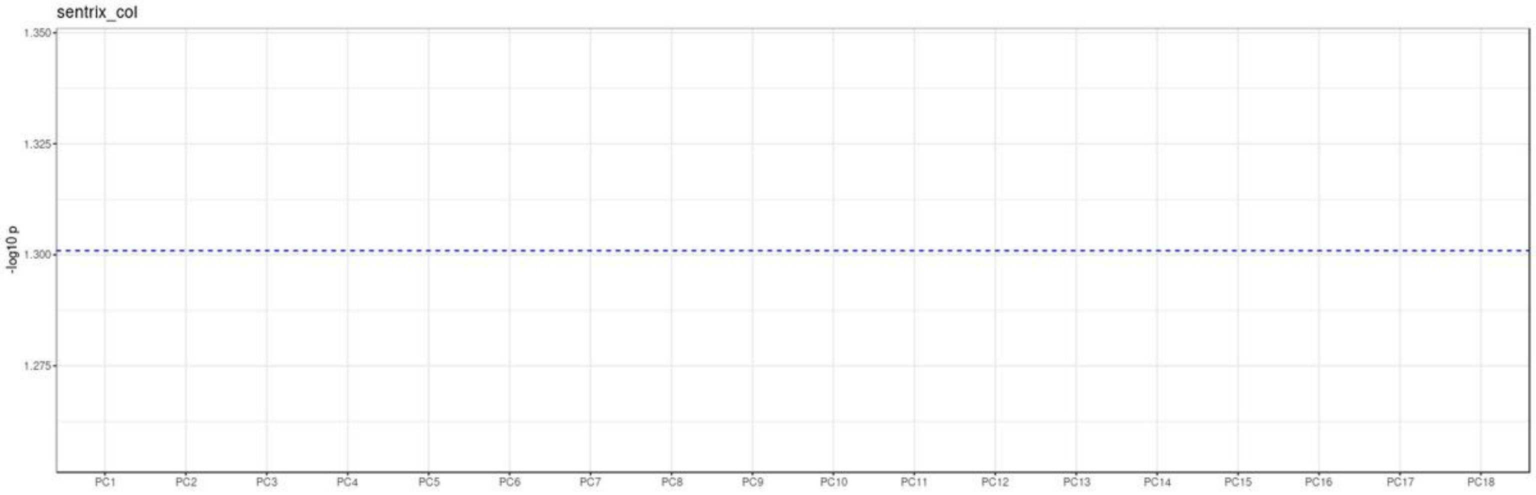

Figure S4b. Normalized probe associations with measured batch variables (sentrix_col). See Figure S4a for details.

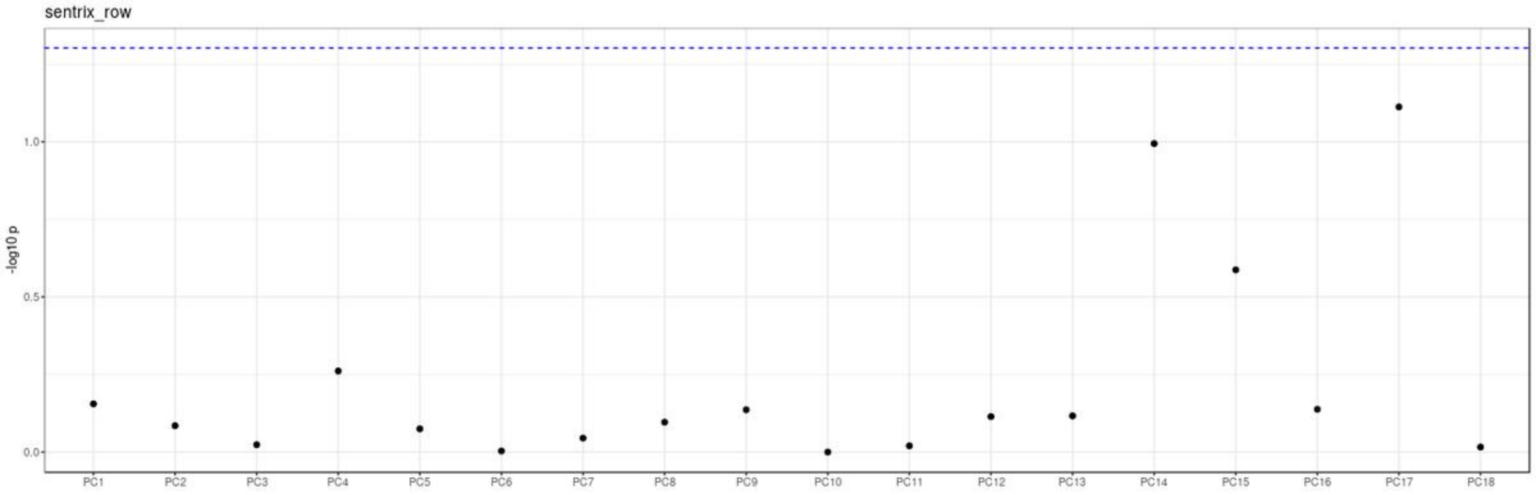

Figure S4c. Normalized probe associations with measured batch variables (sentrix_row). See Figure S4a for details.

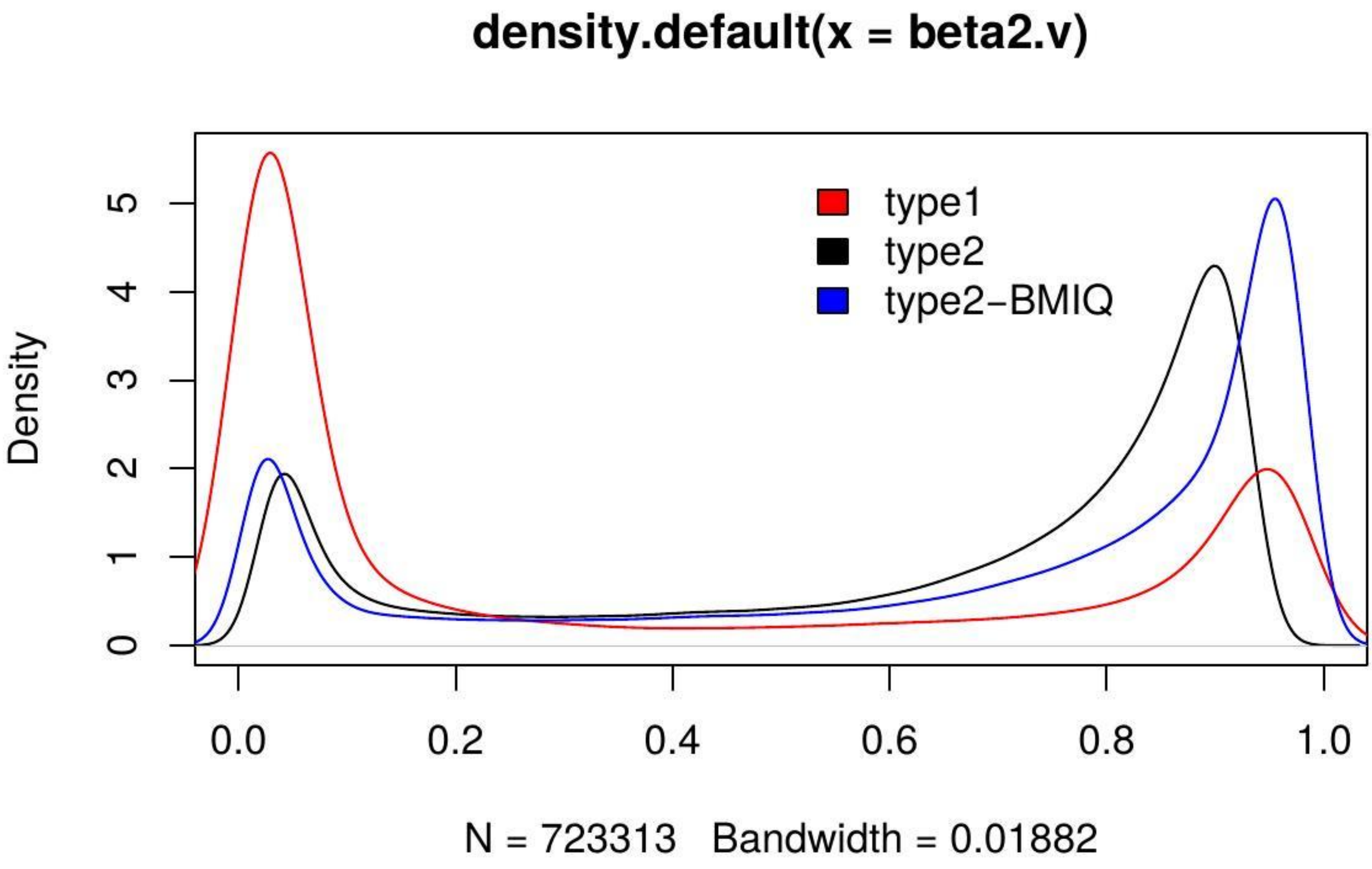

Figure S5. BMIQ probe-design bias correction. Beta-value density distributions for Type 1 probes, Type 2 probes (pre-BMIQ), and Type 2 probes after BMIQ normalization (type2-BMIQ). Successful correction is indicated by alignment of the Type 2 post-BMIQ distribution with the Type 1 distribution.
